# West Nile Encephalitis, an Unusual Infection in a Chronic Lymphocytic Leukemia Patient

**DOI:** 10.1155/2018/3270348

**Published:** 2018-10-14

**Authors:** Manuel R. Espinoza-Gutarra, Sherri L. Cervantez, Zohra Nooruddin

**Affiliations:** ^1^Department of Internal Medicine, UT Health San Antonio, 7703 Floyd Curl Drive, San Antonio, TX 78229, USA; ^2^Department of Hematology-Oncology, Mays Cancer Center, UT Health San Antonio, 7979 Wurzbach Rd., San Antonio, TX 78229, USA; ^3^Department of Hematology-Oncology, South Texas VA, 7400 Merton Minter, San Antonio, TX 78229, USA

## Abstract

CNS involvement by CLL is a rare occurrence, usually happening in the context of a transformation to a more aggressive lymphoma in what is known as Richter's transformation. We report a patient with active CLL who developed confusion and was found to have West Nile encephalitis that initially mimicked CNS involvement by CLL. The patient recovered with supportive treatment and later restarted ibrutinib therapy. This case illustrates the importance of maintaining a broad differential among cancer patients with new onset confusion as well as that of questioning malignant infiltration of CNS when there is concomitant active CNS infection.

## 1. Introduction

Chronic lymphocytic leukemia (CLL) is the most common leukemia in adults; however, central nervous system (CNS) involvement is rare. CLL infiltration of the CNS is often seen with Richter's or prolymphocytoid transformation [1, 2] and is a known, albeit, infrequent complication of CLL with a reported rate of 1-2% [3] with clinically significant disease occurring in only 0.4–0.7% [1]. Higher rates of CNS involvement ranging from 20% to 50% [4, 5] have been reported in autopsy series, but this discrepancy can be explained by inclusion of data on involvement of the spinal cord, the most frequently affected part of the CNS. Far fewer data are available regarding transient CNS infiltration by CLL in the setting of an acute infection.

West Nile virus is a member of the single-stranded RNA Flavivirus family, a member of the Japanese encephalitis virus antigenic complex, which has peak incidence from late August to early September [6]. West Nile virus infection, particularly in its neuroinvasive presentation, is an emerging disease in North America, with increasing rates of morbidity and mortality since an initial outbreak in 1999 and with annual rates of over 1000 cases of neuroinvasive disease over the last decade [7]. Prompt clinical suspicion and recognition of this condition would benefit patients living in these new endemic areas, as migration and climate change will likely increase its incidence [8].

West Nile virus encephalitis is usually asymptomatic; however, it is known to cause clinically significant disease in immunocompromised hosts including severe persistent infection with fatal outcomes [9]. In rare instances, it has been shown to produce clinically significant CNS illness in patients with CLL with poor outcomes [10]. In the case reported by Hollander et al., a patient with known CLL developed acute altered mental status and proceeded to deteriorate clinically; serology studies were negative, but West Nile virus was found on brain tissue during autopsy without evidence of neoplastic infiltration or biologic transformation. Similar to other viral infections, it is characterized by lymphocytosis on CSF studies with normal or slightly elevated protein and normal glucose.

## 2. Case Report

**Figure 1:**
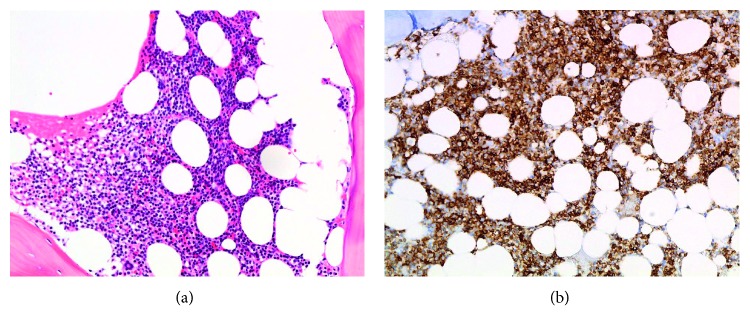
(a) H&E shows infiltrate of small lymphocytes ×50. (b) Immunohistochemistry of CD20 highlighting the lymphocytes ×50.

**Figure 2:**
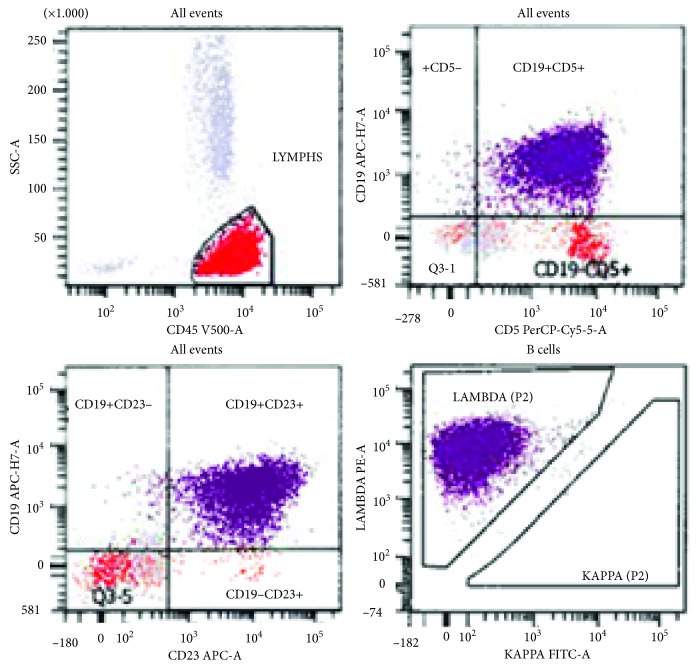
CSF flow cytometry.

We present a 64-year-old Hispanic male with Rai Stage II, IgH mutated, and trisomy 12 positive CLL, diagnosed 13 years prior to admission. He was initially observed for 3 years and then received first-line fludarabine, cyclophosphamide, and rituximab (FCR) for 3 cycles with good response. He remained on observation for 4 additional years and then received an additional 4 cycles of FCR followed by 5 cycles of maintenance rituximab. Five years later, he was found to have 17p deleted recurrent disease and he was started on ibrutinib with good response. He presented to an outside facility with acute onset altered mental status after stopping ibrutinib 2 months earlier due to financial constraints. Cerebrospinal fluid (CSF) analysis was initially concerning for CLL infiltration of the CNS with neoplastic-appearing lymphocytes identified by cytomorphology; however, serological tests for West Nile virus indicated acute infection based on positive IgM and negative IgG; however, PCR could not be performed. The patient received therapy for presumed CNS involvement by CLL with intrathecal methotrexate along with intravenous rituximab and methylprednisolone before being transferred to our institution. On admission, his complete blood count revealed leukocytosis with a normal differential and thrombocytopenia, after reviewing the patients' historical trends, we could discern that the platelet count had been within normal limits in the past and had likely decreased due to the acute illness; additionally, we can appreciate the increasing trend in WBCs until the start of treatment around 10 years prior to this admission (Table 1). Peripheral blood smear showed normal platelet morphology and moderate leukocytosis with lymphocytosis with coarse, block-like chromatin pattern concerning for prolymphocytoid transformation which had not been present previously. Bone marrow biopsy revealed hypercellularity with diffuse infiltrate of the medium to large-sized mononuclear cells with irregular nuclear contours and prominent nucleoli similar to that seen in the peripheral blood smear (Figure 1). Repeat CSF studies at our hospital showed lymphocytes accounting for 49% of total events per flow cytometry, positive for CD19, CD20, CD5, and CD23, with lambda light chain restriction, and negative for CD10 and FMC-7 (Figure 2). The morphology of lymphocytes in CSF was consistent with neoplasia; however, given the admixture of reactive lymphocytes and the presence of known CNS infection, it was deemed that this most likely represented peripheral blood contamination and reactive infiltration of neoplastic lymphocytes.

He was treated supportively during his hospital stay and improved without any further CNS-directed therapy. Ibrutinib was later reinstated after the resolution of thrombocytopenia and BTK mutational testing proved negative. The patient continued to improve during outpatient follow-up, prolymphocitoid morphology disappeared from peripheral smear, and brain MRI showed no evidence of CNS disease.

## 3. Discussion

Previous reports have highlighted the fact that an infectious process can confound the diagnosis, with transmigration of clonal CLL cells being detected even by flow cytometry (FC) and detecting false positives [11, 12]. West Nile virus specifically has been known to cause significant disease in CLL patients, mimicking CNS infiltration, especially because active neoplasm and immunosuppression are the known risk factors for developing the disease as well as having poorer outcomes [13]. In our patient, prompt diagnosis was aided by the fact that he was not on chemotherapy since patients on active rituximab often have negative IgM titers [14].

The differentiation of true CNS infiltration by CLL and peripheral blood contamination or reactive lymphocyte infiltration is usually done through FC analysis. CD49d and CD82 have been proposed as biomarkers that predict CNS infiltration in CLL [15]; however, these studies were not done in our patient. A study by Wu et al. [16] showed that FC was superior to cytomorphology in diagnosing CNS infiltration; however, FC has been shown to be positive for monoclonal lines in the setting of purely nonmalignant conditions or underlying neoplasm without true CNS infiltration [12]. Therefore, cytomorphology still plays a crucial part in expedient clinical decision-making.

Several reports show that patients with true CNS infiltration by CLL have worse outcomes in the setting of prior CLL-directed therapy [1–3, 10]. A previous series identified the need for aggressive systemic and/or intrathecal treatment in the setting of true CLL involvement of CNS [17]. Additionally, presence of prolymphocytoid morphology has been associated with high-risk mutations, Richter's transformation, and overall more aggressive disease [18]. Although our patient received a single dose of chemotherapy, his continued clinical improvement with mere supportive therapy for West Nile encephalitis argued against active CNS infiltration by CLL.

## 4. Conclusion

Our case report highlights the complexities in differentiating West Nile virus encephalitis from CNS involvement by CLL due to the presence of reactive CLL cells in the CSF. This case further exemplifies the need for thorough evaluation with FC, cytopathology, and infectious markers prior to treatment of apparent CLL infiltration of the CNS as reversible infectious etiologies may mimic more serious leukemic transformations.

## Figures and Tables

**Table 1:** Laboratory values.

	Normal range	13 years prior to admission	10 years prior to admission	5 years prior to admission	1 year prior to admission	On admission	On discharge
WBC	4.8–10.8 10^3^/*μ*L	30.4	196.9	113.7	91.9	13	4.3
HCT	42–50%	42.3	40.9	37.6	35.9	28.6	23.9
Hb	14–17 g/dL	14.9	13.3	12.6	11.8	10.1	8.4
RBC count	4.7–6.1	4.67	4.30	4.05	3.83	3.16	2.59
PLT	140–400 10^3^/*μ*L	223	238	150	143	49	36
MCV	80–99 fL	90.6	95.1	92.7	93.7	90.4	92.3

Differential count: percentage (absolute)							
Neutrophils	40–74% (1.9–8 10^3^/*μ*L)	19.3 (5.9)	8.3 (16.4)	10 (11.3)	6.9 (6.4)	50.8 (6.6)	54.5 (2.3)
Lymphocytes	19–48% (0.9–5.2 10^3^/*μ*L)	75 (22.8)	91.1 (179.4)	64 (72.7)	90.8 (83.4)	44.8 (5.8)	37.7 (1.6)
Atypical lymphocytes	0–10%	6% + smudge cells	22% + smudge cells	20% and 2% blasts	27%	2%	3.1%
Monocytes	3.4–9% (0.16-1 10^3^/*μ*L)	4.1 (1.2)	0.5 (0.9)	3 (3.4)	1.5 (1.3)	3 (0.4)	2.1 (0.1)
Eosinophils	0–7% (0–0.8 10^3^/*μ*L)	1.4 (0.4)	0 (0.1)	1 (1.1)	0.5 (0.5)	0.4 (0.1)	5.6 (0.2)
Basophils	0–1.5% (0–0.2 10^3^/*μ*L)	0.2 (0.1)	0.1 (0.2)	2 (2.2)	0.3 (0.3)	1 (0.1)	0.1 (0.0)
IgA	85–385 mg/dL	128			23		
IgG	564–1765 mg/dL	1130			489		
IgM	45–250 mg/dL	121			28		
LDH	98–192 IU/L		154	225	377	261	
